# Supplementary material for: Predicting Keratoconus Progression and Need for Corneal Crosslinking Using Deep Learning
Source: J Clin Med. 2021 Feb 18;10(4):844. doi: 10.3390/jcm10040844 (PMC7923054; doi:10.3390/jcm10040844)
Supplement: Supplementary file 1 [file jcm-10-00844-s001.zip]

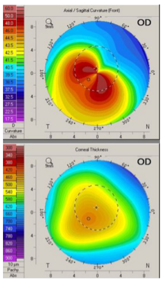

01.jpg

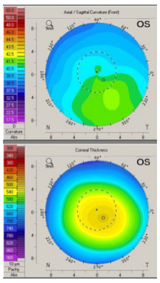

02.jpg

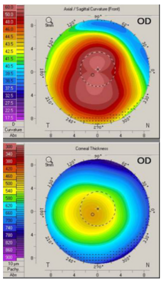

03.jpg

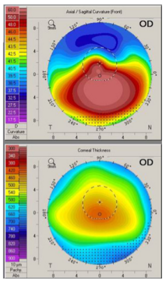

05.jpg

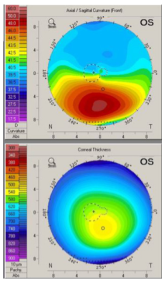

06.jpg

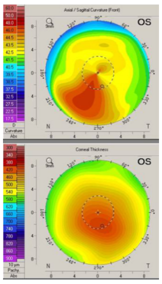

08.jpg

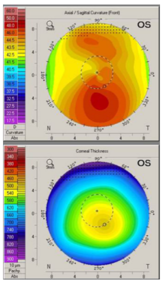

09.jpg

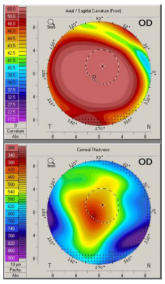

10.jpg

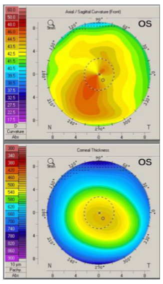

11.jpg

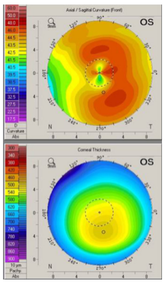

12.jpg

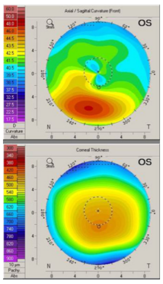

13.jpg

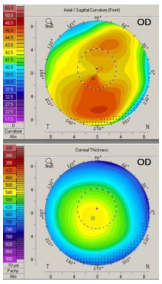

14.jpg

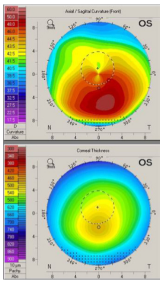

15.jpg

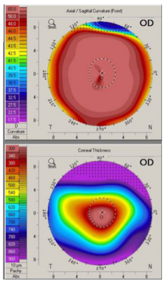

16.jpg

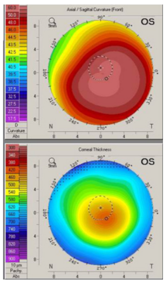

17.jpg

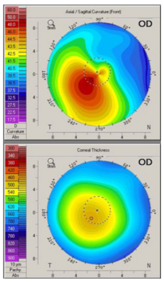

18.jpg

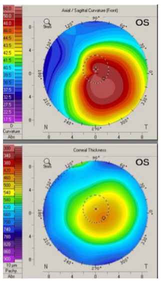

19.jpg

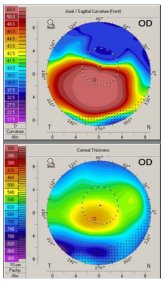

20.jpg

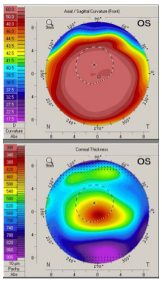

21.jpg

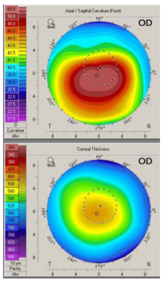

22.jpg

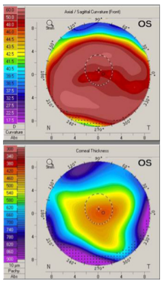

23.jpg

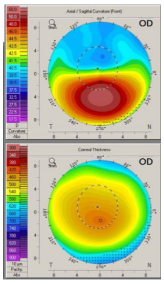

24.jpg

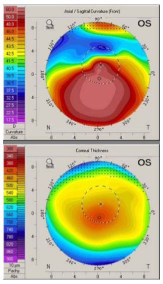

25.jpg

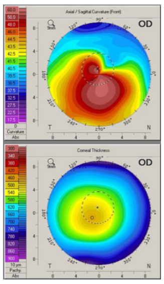

26.jpg

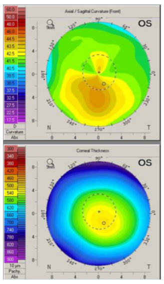

27.jpg

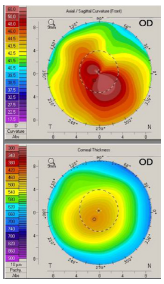

28.jpg

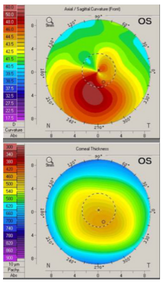

29.jpg

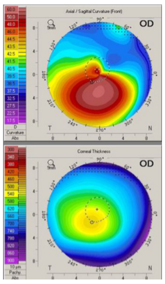

30.jpg

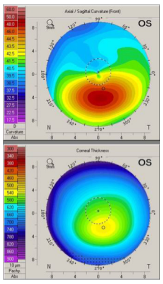

31.jpg

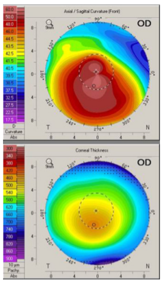

32.jpg

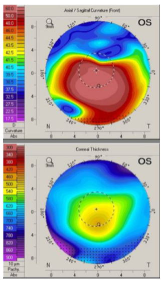

33.jpg

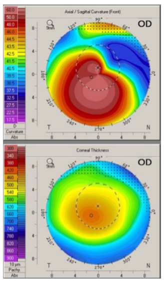

34.jpg

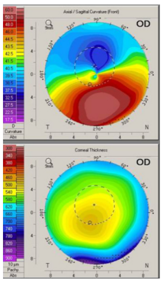

35.jpg

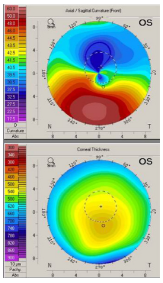

36.jpg

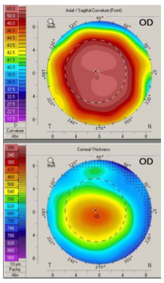

37.jpg

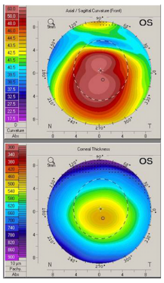

38.jpg

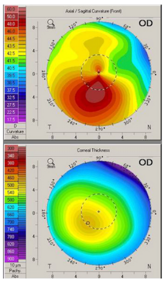

39.jpg

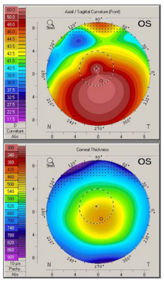

40.jpg

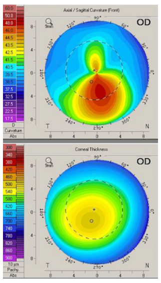

41.jpg

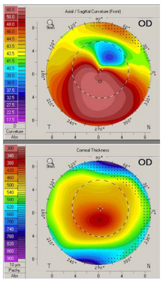

42.jpg

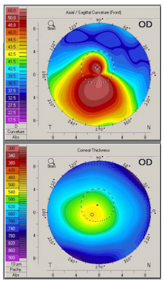

43.jpg

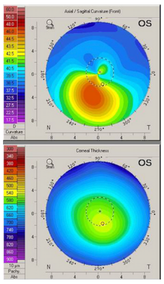

44.jpg

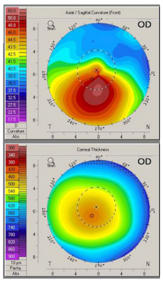

45.jpg

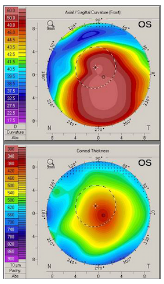

46.jpg

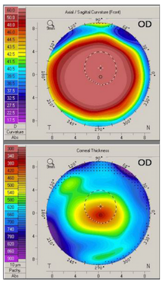

47.jpg

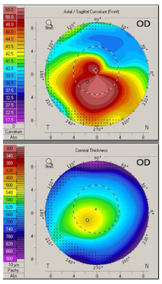

48.jpg

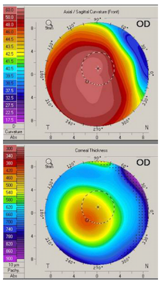

49.jpg

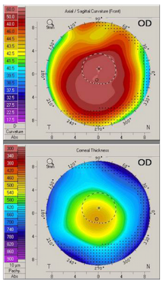

52.jpg

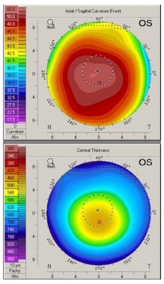

53.jpg

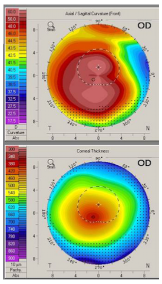

54.jpg

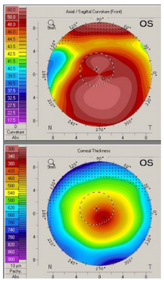

55.jpg

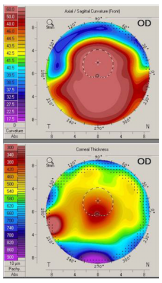

56.jpg

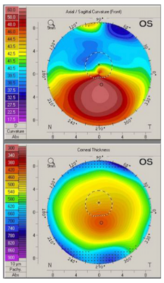

57.jpg

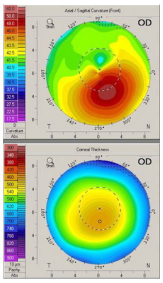

58.jpg

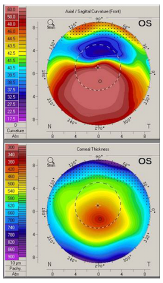

59.jpg

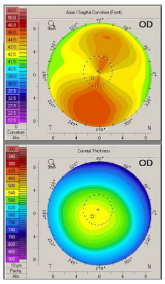

60.jpg

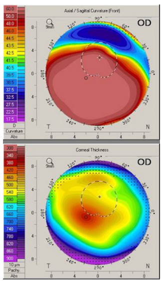

61.jpg

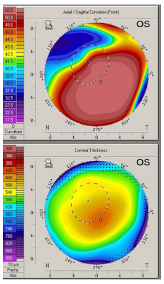

62.jpg

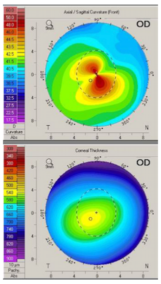

63.jpg

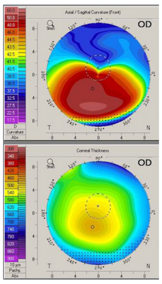

64.jpg

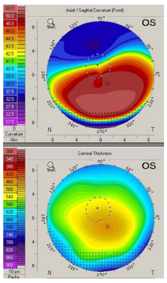

65.jpg

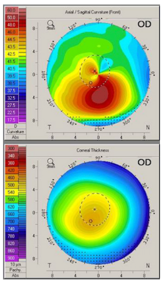

66.jpg

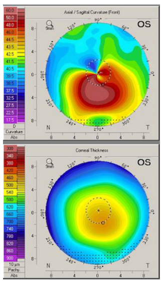

67.jpg

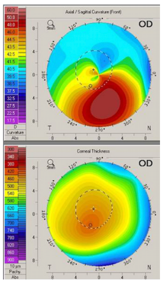

68.jpg

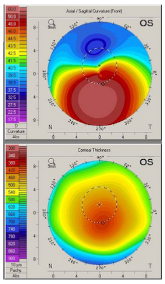

69.jpg

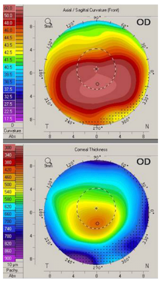

70.jpg

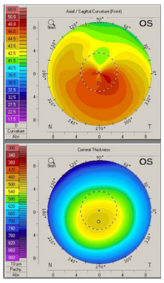

71.jpg

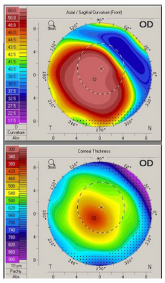

72.jpg

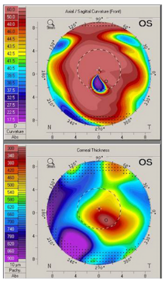

73.jpg

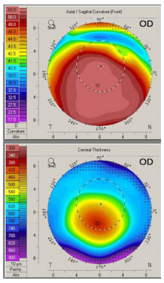

74.jpg

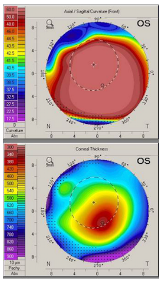
75.jpg
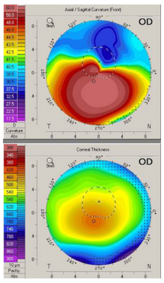
76.jpg
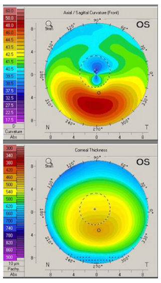
77.jpg
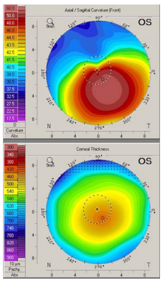
78.jpg
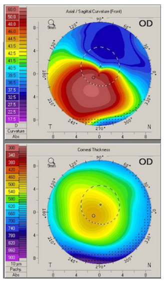
79.jpg
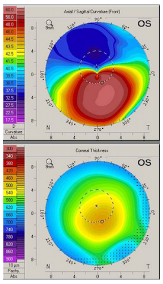
80.jpg
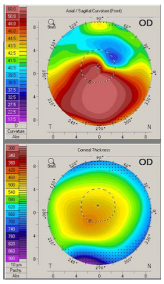
81.jpg
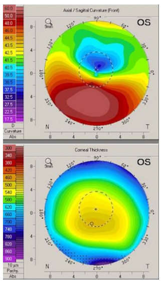
82.jpg
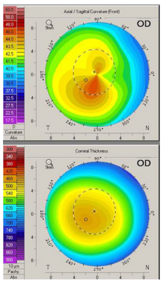
83.jpg
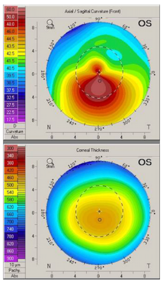
84.jpg
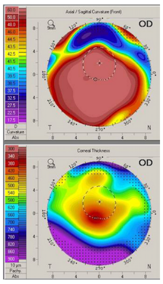
85.jpg
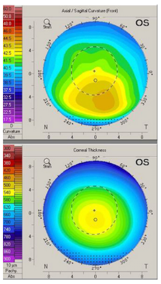
86.jpg
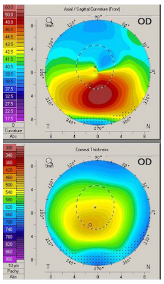
87.jpg
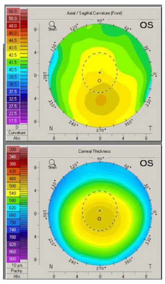
88.jpg
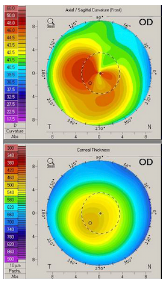
89.jpg
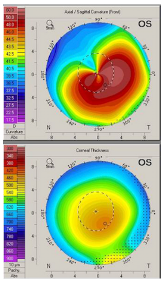
90.jpg
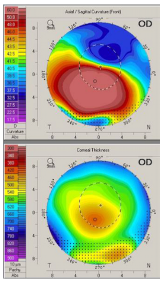
91.jpg
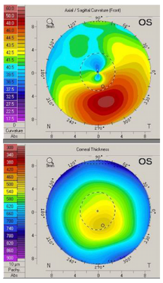
92.jpg
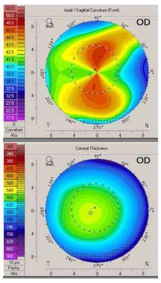
93.jpg
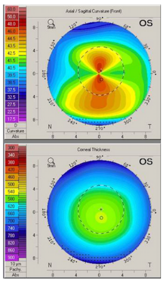
94.jpg
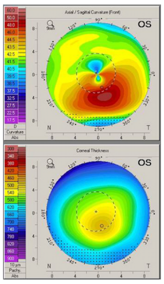
95.jpg
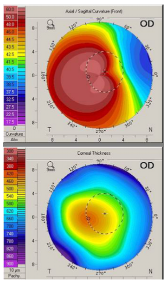
96.jpg
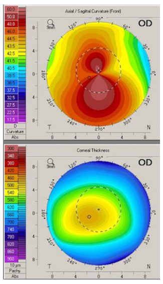
104.jpg
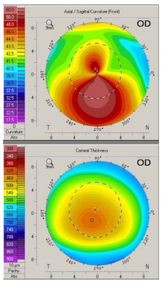
106.jpg
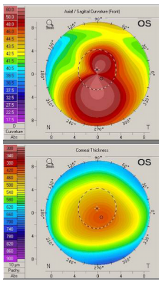
107.jpg
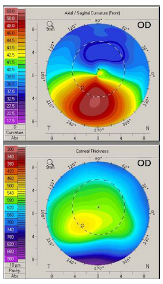
108.jpg
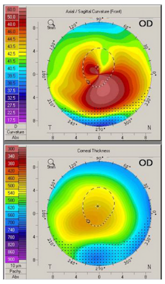
110.jpg
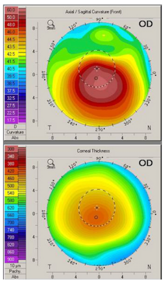
111.jpg
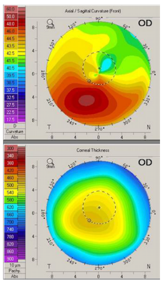
112.jpg
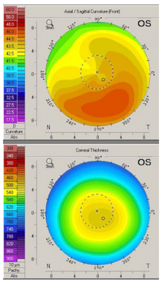
113.jpg
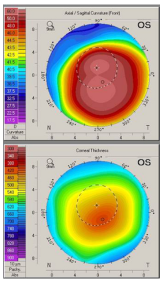
114.jpg
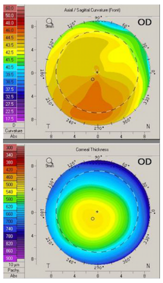
115.jpg
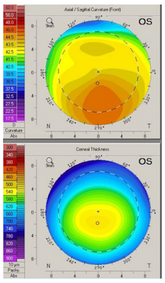
116.jpg
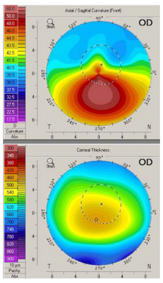
117.jpg
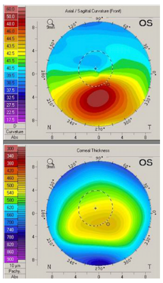
118.jpg

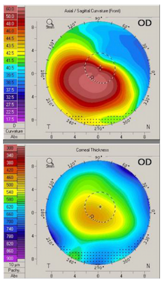
119.jpg

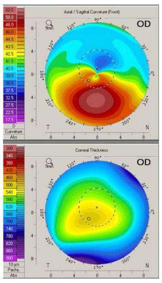
120.jpg

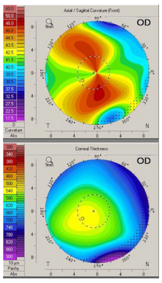
122.jpg

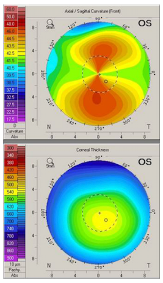
123.jpg

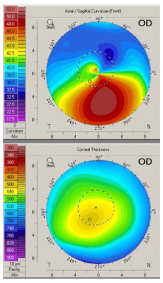
124.jpg

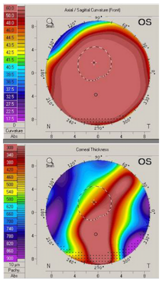
125.jpg

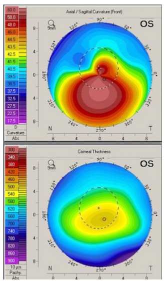
126.jpg

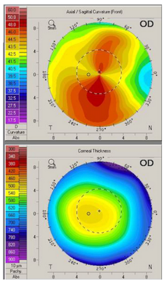
127.jpg

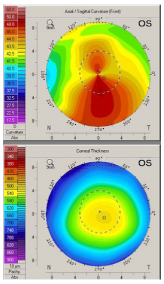
128.jpg

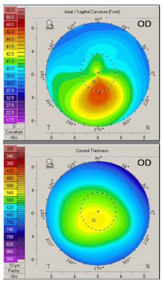
129.jpg

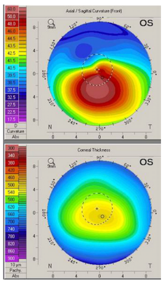
130.jpg

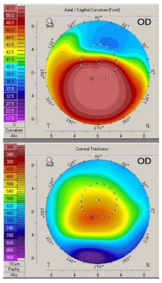
131.jpg

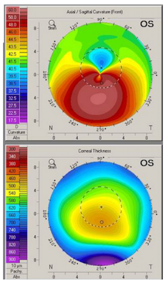
132.jpg

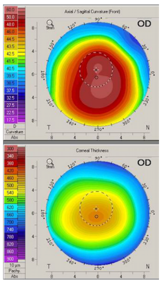
133.jpg

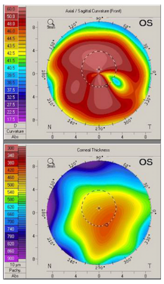
134.jpg

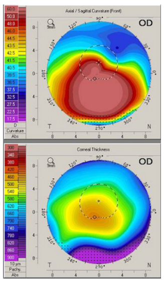
135.jpg

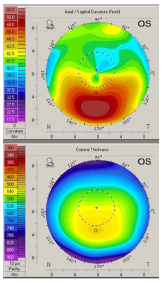
136.jpg

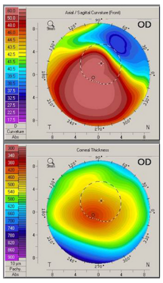
137.jpg

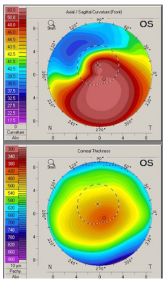
138.jpg

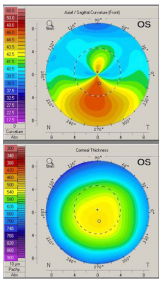
139.jpg

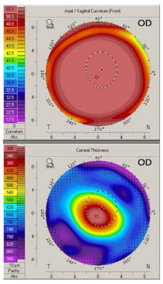
140.jpg

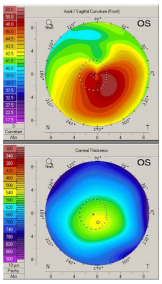
141.jpg

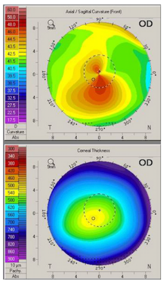
142.jpg

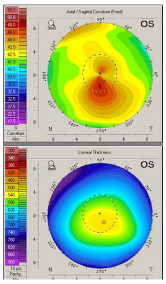
143.jpg

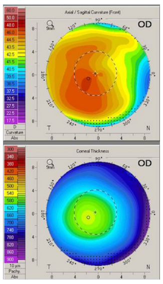
144.jpg

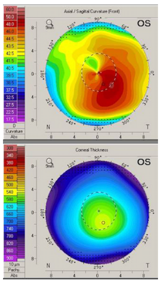
145.jpg

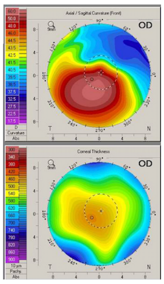
146.jpg

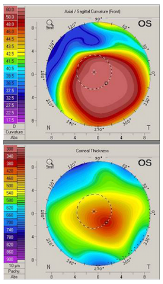
147.jpg

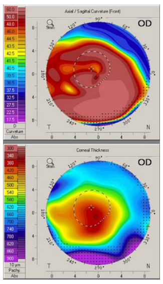
148.jpg

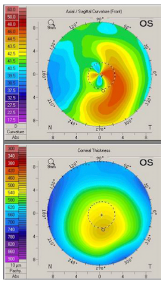
149.jpg

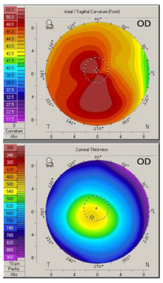
150.jpg

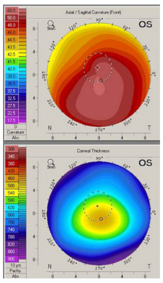
151.jpg

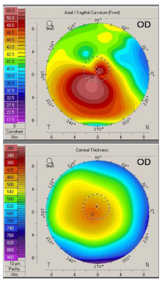
152.jpg

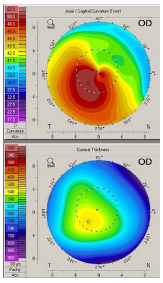
154.jpg

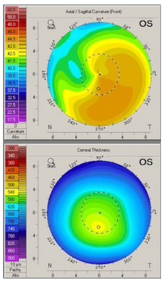
155.jpg

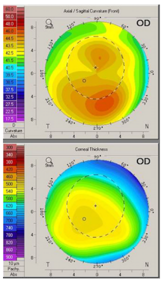

156.jpg

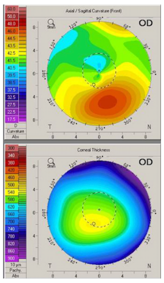

157.jpg

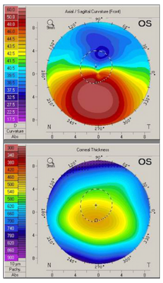

158.jpg

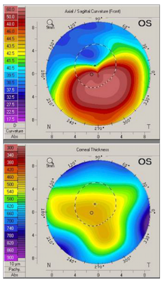

159.jpg

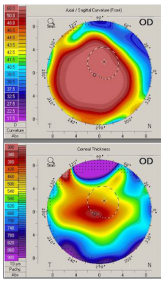

160.jpg

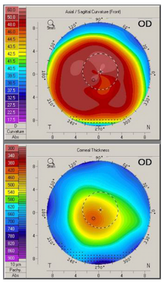

161.jpg

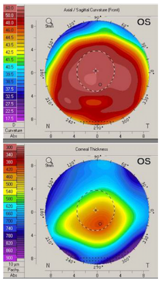

162.jpg

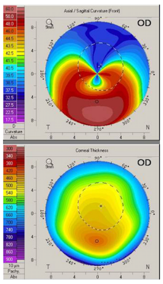

163.jpg

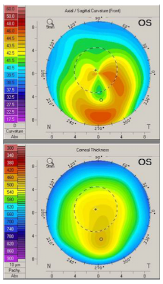

164.jpg

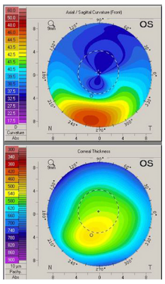

165.jpg

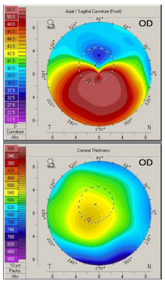

166.jpg

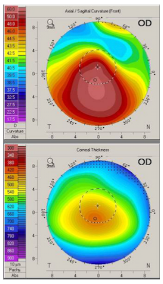

167.jpg

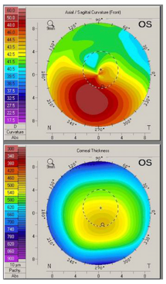

168.jpg

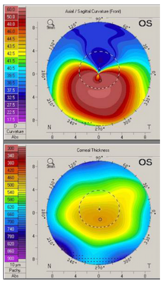

169.jpg

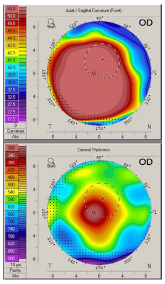

170.jpg

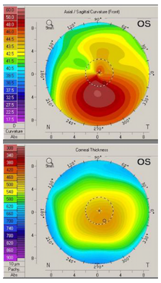

171.jpg

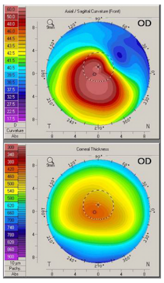

172.jpg

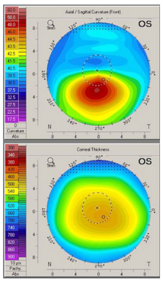

173.jpg

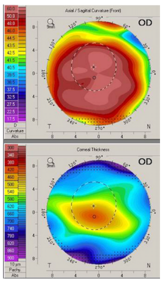

174.jpg

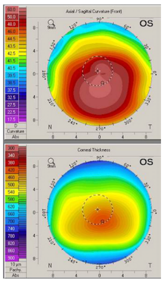

175.jpg

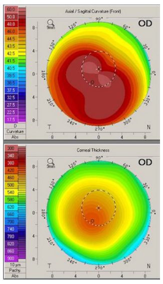

176.jpg

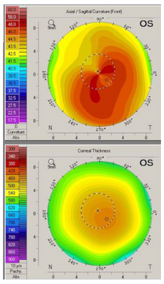

177.jpg

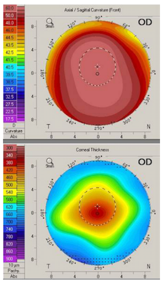

178.jpg

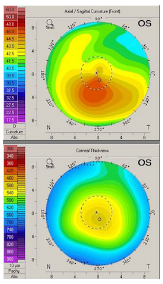

179.jpg

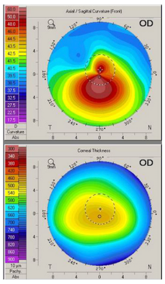

180.jpg

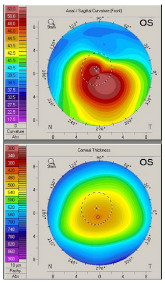

181.jpg

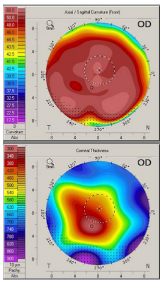

182.jpg

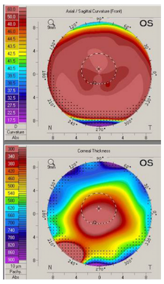

183.jpg

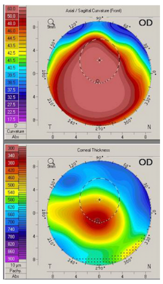

184.jpg

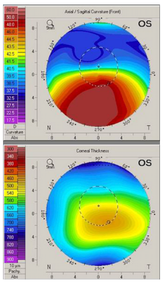

185.jpg

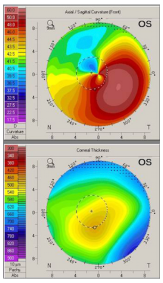

186.jpg

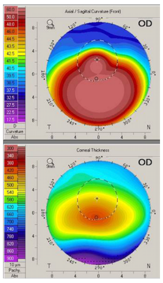

187.jpg

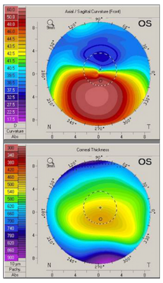

188.jpg

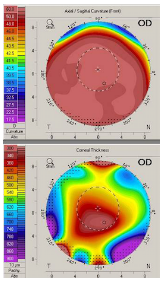

189.jpg

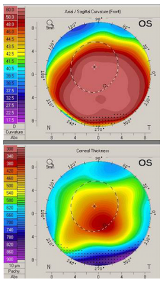

190.jpg

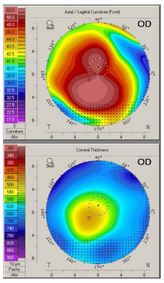
191.jpg
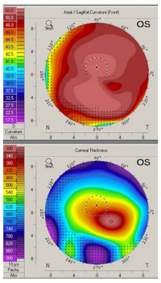
192.jpg
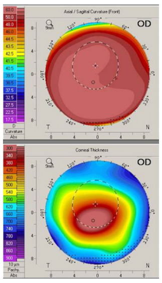
193.jpg
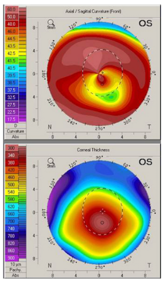
194.jpg
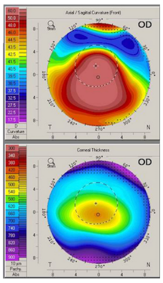
195.jpg
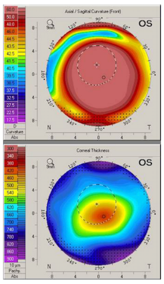
196.jpg
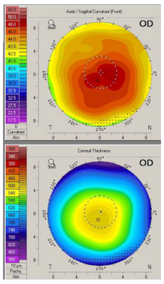
197.jpg
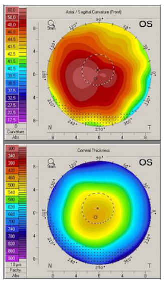
198.jpg
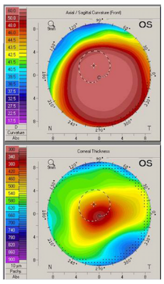
200.jpg
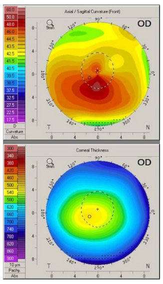
201.jpg
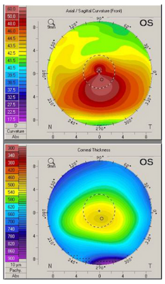
202.jpg
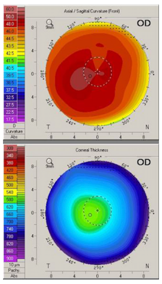
203.jpg
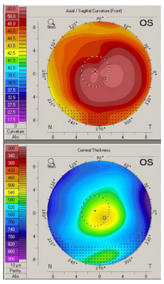
204.jpg
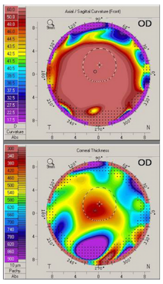
205.jpg
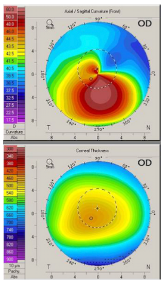
207.jpg
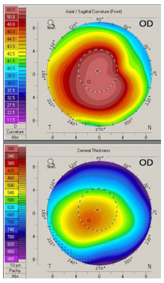
208.jpg
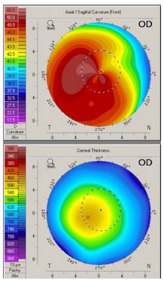
209.jpg
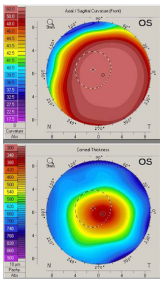
210.jpg
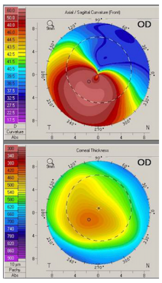
211.jpg
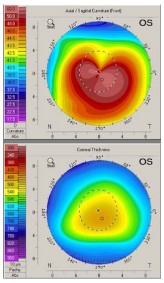
212.jpg
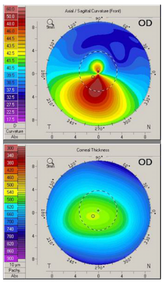
213.jpg
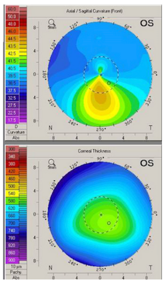
214.jpg
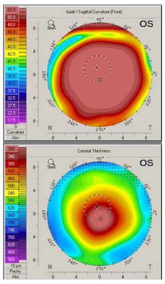
215.jpg
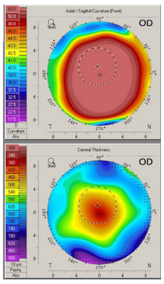
216.jpg
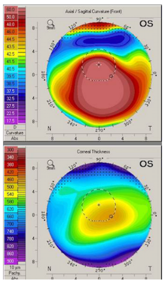
217.jpg
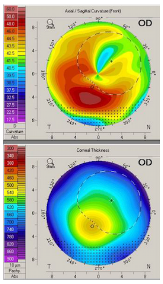
218.jpg
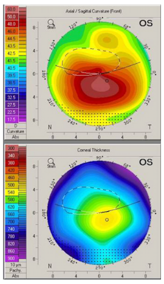
219.jpg
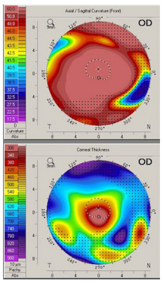
220.jpg
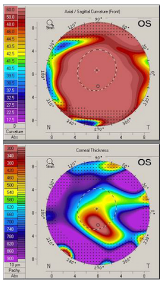
221.jpg
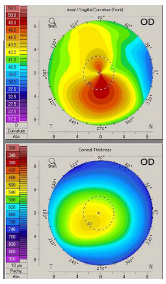
222.jpg
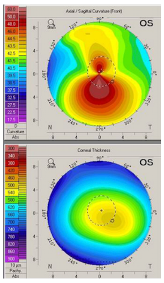
223.jpg
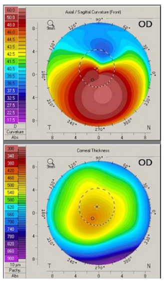
224.jpg
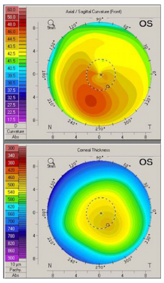
225.jpg
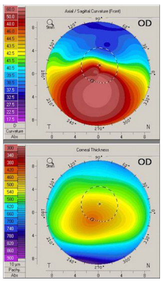
226.jpg
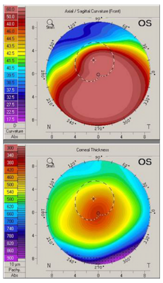
227.jpg

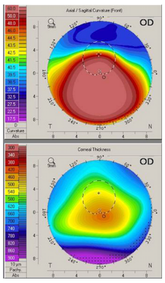

228.jpg

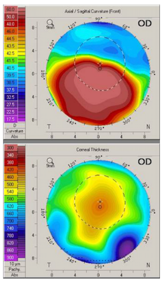

229.jpg

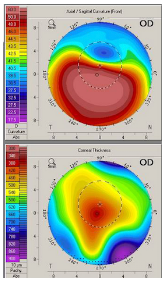

230.jpg

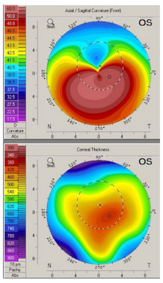

231.jpg

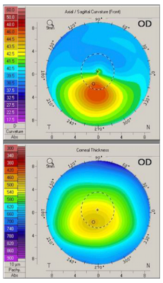

232.jpg

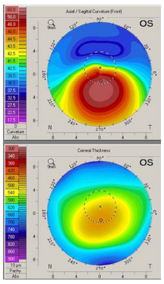

233.jpg

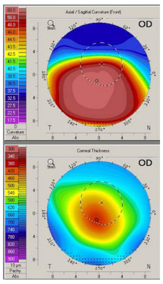

234.jpg

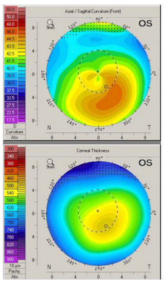

235.jpg

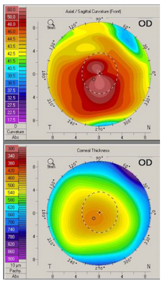

236.jpg

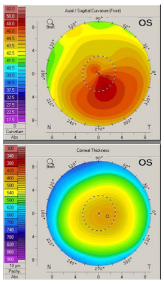

237.jpg

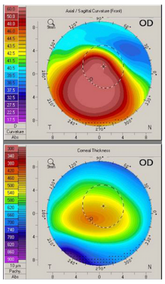

238.jpg

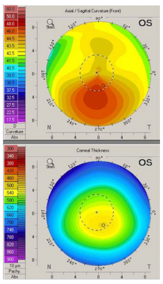

239.jpg

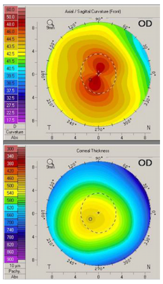

240.jpg

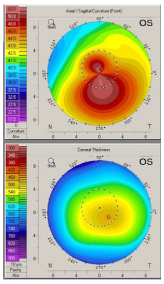

241.jpg

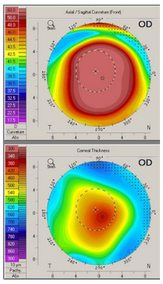

242.jpg

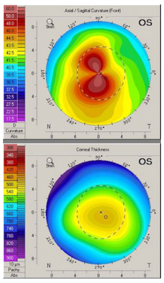

243.jpg

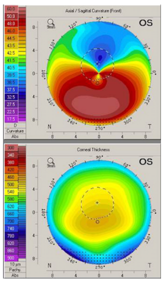

244.jpg

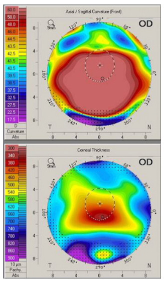

245.jpg

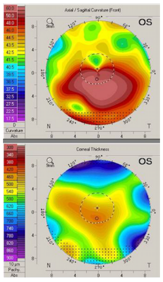

246.jpg

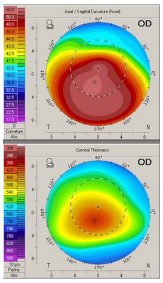

247.jpg

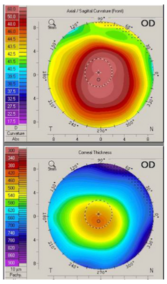

249.jpg

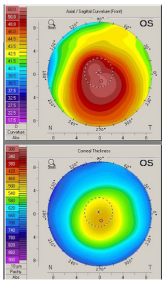

248.jpg

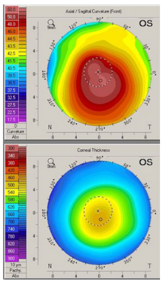

250.jpg

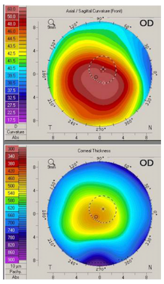

251.jpg

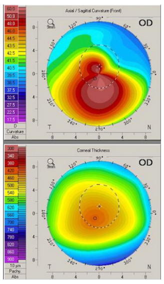

252.jpg

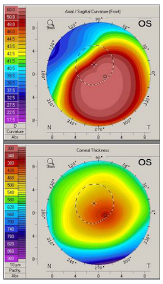

253.jpg

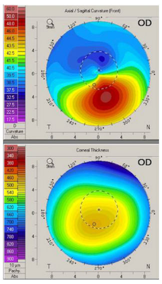

254.jpg

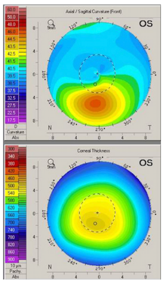

255.jpg

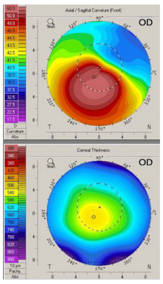

256.jpg

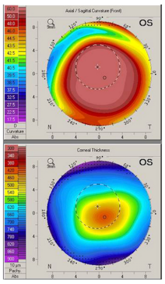

257.jpg

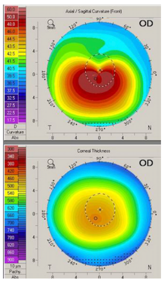

258.jpg

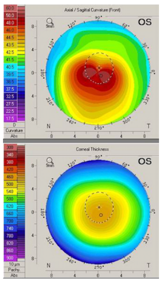

259.jpg

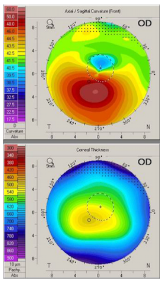

261.jpg

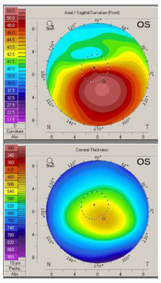

262.jpg

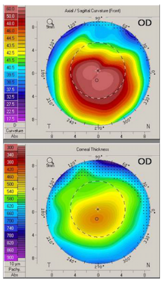

263.jpg

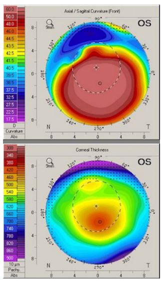
264.jpg

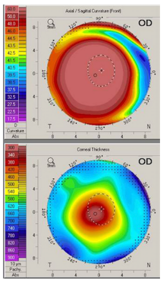
265.jpg

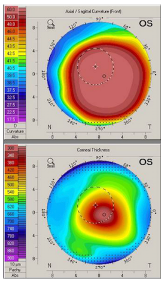
266.jpg

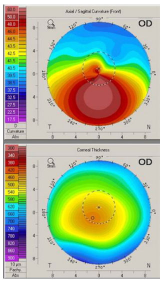
268.jpg

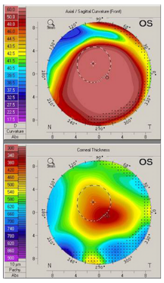
269.jpg

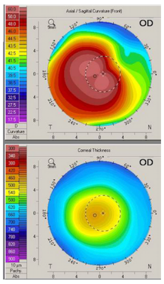
270.jpg

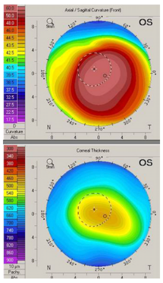
271.jpg

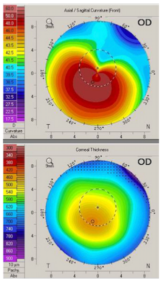
272.jpg

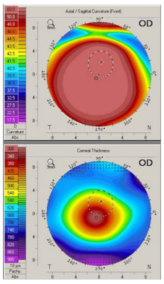
273.jpg

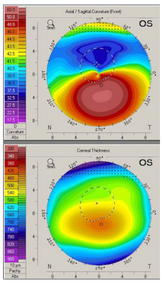
274.jpg

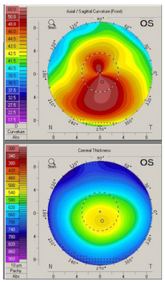
04.jpg

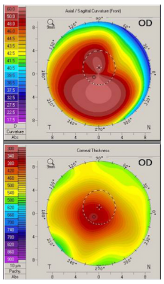
07.jpg

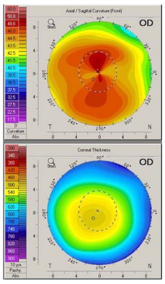
50.jpg

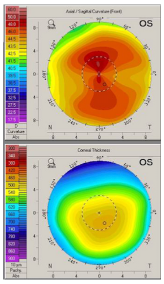
51.jpg

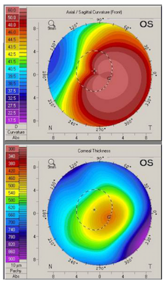
97.jpg

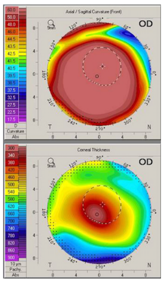
98.jpg

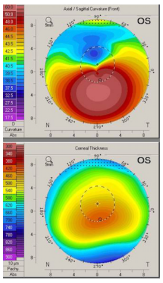
99.jpg

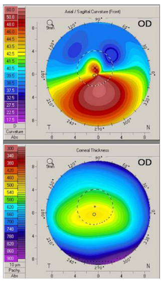
100.jpg

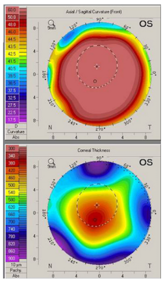
101.jpg

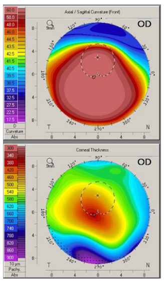
102.jpg

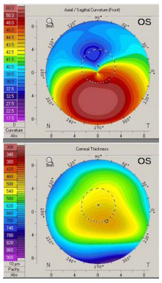
103.jpg

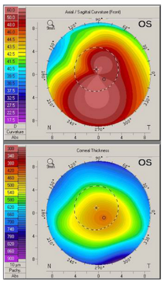
105.jpg

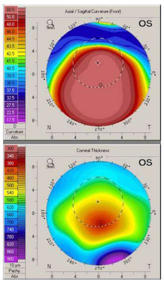
109.jpg

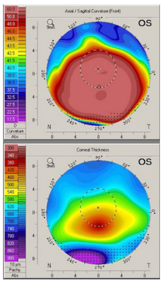
121.jpg

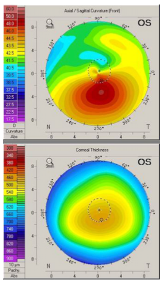
153.jpg

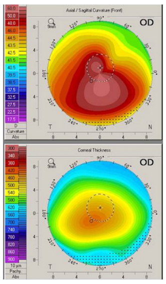
199.jpg

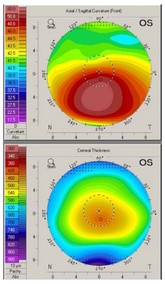
206.jpg

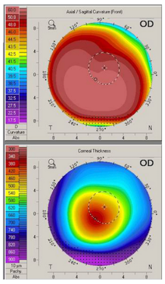
260.jpg

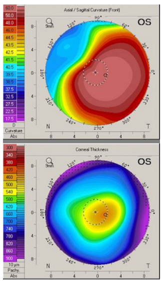
267.jpg
